# The first influenza pandemic of the 21st century

**DOI:** 10.4103/0256-4947.59365

**Published:** 2010

**Authors:** Sami Al Hajjar, Kenneth McIntosh

**Affiliations:** a From the King Faisal Specialist Hospital & Research Centre, Riyadh, Saudi Arabia; b From the Harvard Medical School, Boston, Massachusetts, USA

## Abstract

The 2009 H1N1 influenza virus (formerly known as swine flu) first appeared in Mexico and the United States in March and April 2009 and has swept the globe with unprecedented speed as a result of airline travel. On June 11, 2009, the World Health Organization raised its pandemic level to the highest level, Phase 6, indicating widespread community transmission on at least two continents. The 2009 H1N1 virus contains a unique combination of gene segments from human, swine and avian influenza A viruses. Children and young adults appear to be the most affected, perhaps reflecting protection in the elderly owing to exposure to H1N1 strains before 1957. Most clinical disease is relatively mild but complications leading to hospitalization, with the need for intensive care, can occur, especially in very young children, during pregnancy, in morbid obesity, and in those with underlying medical conditions such as chronic lung and cardiac diseases, diabetes, and immunosuppression. Bacterial coinfection has played a significant role in fatal cases. The case of fatality has been estimated at around 0.4%. Mathematical modeling suggests that the effect of novel influenza virus can be reduced by immunization, but the question remains: can we produce enough H1N1 vaccine to beat the pandemic?

With the emergence of the second wave of the 2009 influenza A (H1N1) virus, there have been concerns that this pandemic may rival those of 1957, 1968, and even 1918 in which not thousands, but millions of people around the world died from the disease (Table 1).^1^,^2^ WHO is advising countries of the northern and southern hemispheres to prepare for a second wave of H1N1, in which large numbers of severely ill patients requiring more and more intensive care infrastructure are likely to be seen, creating pressures that could overwhelm hospitals and intensive care units and possibly disrupt the provision of care of other diseases. The newly developed H1N1 vaccine is expected to reduce the impact of the second wave of H1N1 influenza in the population, especially on high-risk groups, with diminished complications, hospitalization rates and mortality. On the other hand, previous H1N1 strains have developed antiviral resistance, and this, as well as mutation to greater virulence, remain concerns for the future.^3^,^4^ Past pandemics were characterized by several features that we have seen since March, 2009: the rapid spread of a virus with novel antigenic determinants; a change in pathogenicity with high death rates in younger age groups; successive pandemic waves; apparent higher transmissibility than that of the seasonal influenzas; and differences in impact in different geographic region.^5^ The overall mortality in the previous century's three pandemics ranged from 1 million to more than 45 million deaths.^2^,^6^ In the three previous influenza pandemics, vaccines were not produced in time to have any substantial impact.^7^ Even though the technology of vaccine manufacture (production in embryonated eggs) has changed little since the 1930's, there is some hope that vaccines will be available to mitigate the force of later waves of the current epidemic. In addition, several clinically useful antiviral drugs are now available, although there are still concerns about development of resistance.^3^

**Table 1:** Influenza pandemic of the 20th century.

Date	Strain	Estimate number of worldwide deaths	Comments
1918-1919 (Spanish Flu)	H1N1	Over 50 million	Three waves: A first, mild wave in the spring of 1918 was replaced by a second wave in September to November, 1918 that resulted in a mortality rate of over 2.5%. A third wave with equally high mortality rates swept around the world in 1919. The virus probably originated from the United States and then spread to Europe.

1957-1958 (Asian Flu)	H2N2	1-1.5 million	Two waves: The virus originated in Southern China in February 1957 and spread over 3 months to Singapore, Hong Kong and Japan and in October 1957 reached the United Kingdom and United States. A second wave was detected in January 1958.

1968-1969 (Hong Kong Flu)	H3N2	¾ million	Two waves in the winters of 1968-1969 and 1969-1970. The virus originated from Hong Kong in July 1968.

## Influenza virus: Back to basics

The viruses that cause influenza (influenza A, B, and C) belong to the family Orthomyxoviridae, which is characterized by a segmented minus-strand RNA genome. Influenza A and B viruses genomes consist of 8 separate segments. These include the following: three transcriptases (PB1, PB2, and PA), two surface glycoproteins, the hemagglutunin (H or HA) and neuramidase (N and NA), two matrix proteins (M1 and M2), and one nucleocapsid protein (NP). Epidemic disease is caused by influenza viruses type A and B. Type C influenza viruses cause sporadic mild influenza-like illness in children. The focus of this article will be on influenza A virus, which may infect humans and birds and most importantly has the capability of developing into pandemic virus.^8^ Type A influenza has been divided into multiple subtypes, and the natural host for most of these are various avian species. In addition, influenza A viruses of a few distinct subtypes have been isolated from pigs, horses, seals, whales and human beings. The genome of the virus codes for two important surface glycoproteins, the hemagglutinin (H or HA) and the neuraminidase (N or NA). Based on both sequence and antigenic analysis, sixteen distinct H (H1-H16) and nine distinct N (N1-N9) subtypes are now recognized in animal and avian influenza viruses, but only 3 H subtypes (H1, H2 and H3) and 2 N subtypes (N1, N2) have caused extensive outbreaks in human beings.^9^ The influenza virus has a poor ability to proofread its genetic material while replicating, which results in frequent errors in progeny genes, and thus frequent mutations. When such minor changes occur in the H and N proteins they result in “antigenic drift,” the slow but significant change in antigenicity that occurs over time in both influenza A and influenza B and that requires periodic changes in the yearly vaccine.^10^ An example of such drift occurred during the 2003/2004 influenza season when the H3N2 circulating virus developed over 80% drift from the virus that was used to make one of the three major vaccine components that year (Table 2). Further, marked changes in H, with or without similar changes in N, termed “antigenic shift”^11^ occur when new H or N gene segments are acquired by a process known as “reassortment.” This may take place by the mixing of genetic segments during dual infection of cells by a human and an animal virus. When such viruses containing reassorted gene segments are introduced into a population that has no pre-existing immunity, they may lead to a pandemic. This happened in 1957 and 1968.^10^,^11^

**Table 2:** Antigenic drift and shift.

Drift	Shift
Minor change within subtype	Major change, new subtype
Point mutations	Exchange of gene segments
Occurs in A and B subtypes	Occurs in A subtypes only
May cause epidemics	May cause pandemic
Example: A/Fujian (H3N2) replaced A/Panama (H3N2) in 2003-2004	Example: H3N2 replaced H2N2 in 1968

Devastating pandemics take place when populations are exposed to a new viral subtype in the absence of pre-existing immunity. The infectious capabilities of a new virus that emerges in this way through reassortment are likely to be acquired from one or more of the human influenza gene segments. Conditions favorable for the emergence of an antigenic shift (reassortment) involve humans living in close proximity to domestic poultry and pigs.^12^ Pigs play an important role in interspecies transmission of influenza virus. Susceptible pig cells process receptors for both avian and human influenza strains which allow the pigs to serve as mixing vessels for the exchange of genetic material between human and avian viruses resulting in the appearance of novel subtypes. Analysis of the 1957 H2N2 pandemic strain found that the emergent virus resulted from the acquisition by previously circulating human H1N1 of three new gene segments of avian origin (the H2 gene, the N2 gene, and one other). Similarly, the 1968 pandemic H3N2 virus acquired two new genes from an avian virus closely related to viruses isolated from ducks in Asia in 1963. In contrast, the 1918 H1N1 virus appears to have been an avian-like influenza virus derived in toto from an unknown source.^13^,^14^ The currently circulating novel influenza H1N1 viruses that have been isolated around the globe during 2009 appear to have originated from two unrelated swine viruses, one of them a derivative of the 1918 human virus.^15^

## Evolution, zoonotic transmission and possible origin of 2009 H1N1 (Swine influenza)

**Figure 1:**
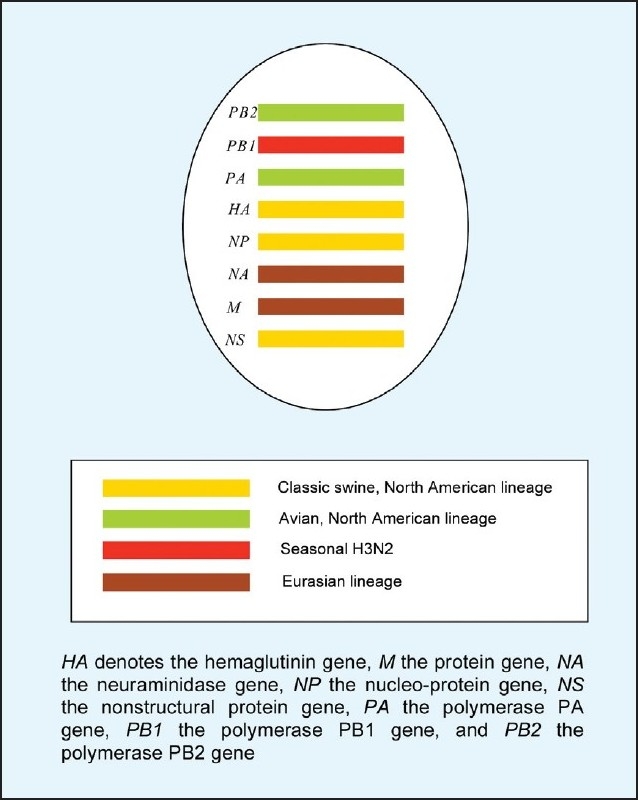
2009 influenza A (H1N1) virus genotype.

The 1918 H1N1 pandemic is believed to have also affected swine at that time. Its descendents have been enzootic in pigs up ever since (Table 3).^15^,^16^ The first influenza A isolated from diseased pigs in the United States (USA) was in 1930.^17^ These H1N1 swine viruses are called the classical swine H1N1 viruses and have continued to circulate in pigs in the Americas, Asia and, until 1980, also in Europe, and they remain relatively antigenically stable.^18^ This swine H1N1 subtype has crossed over to humans periodically, including the Fort Dix outbreak in 1976,^19^ resulting in infections that have been occasionally fatal, particularly in pregnant or immunocompromised persons, but not producing human epidemics. Moreover, following the human pandemic of the H3N2 subtype in 1968, H3N2 influenza virus infected pigs although such porcine strains have shown less antigenic drift in swine than in humans.^20^ In 1998, H3N2 viruses with genes derived from human, swine and avian genes of North America (“triple reassortant viruses”) were first isolated from pigs in the USA.^21^ The triple reassortant H3N2 viruses also continue to acquire other virus genes via reassortment to generate triple reassortant H1N2 or H1N1 viruses.^22^ Swine viruses of subtypes H1N1, H1N2 and H3N2 have been reported to cause occasional human infection during this time.^23^ Between 1958 and 2005, 37 human swine-origin influenzas were reported. Twenty-two (51%) of these cases reported recent exposure to pigs. The overall fatality rate was 17%.^24^ Prior to the current pandemic, but after December 2005, eleven sporadic cases of triple reassortant H1 viruses were reported to the Centers for Disease Control and Prevention (CDC) in the USA, ten carrying H1N1 genes and one H1N2 genes. Some of the patients had close exposure to pigs. Possible limited human-to-human transmission was reported in several situations.^25^ Genetic analysis of 2009 H1N1 viruses isolated in North America, Europe and Asia revealed quadruple reassortant swine influenza A viruses that have not been recognized previously in pigs or humans. The virus resulted from the reassortment of North American H3N2 and H1N2 swine viruses (triple reassortment viruses: avian/swine/human with Eurasian swine viruses).^15^,^26^–^28^ Sequence analysis also suggests that PB2 and PA genes originated from American H3N2 avian virus; a PB1 originated from H3N2: HA, NP, and NS genes originated from classical swine virus: and NA and M genes originated from Eurasian swine virus (Figure 1). One of swine genes of this new virus derived from the 1918 human virus, so the strain causing the 2009 pandemic is a fourth generation descendant of the 1918 virus.^15^ The 2009 H1N1 viruses are more pathogenic in mammalian models than seasonal H1N1 viruses, showing the ability to replicate and cause appreciable pathology in the lungs of mice, ferrets and non-human primates. The pathologic changes seen were similar to those found in the lungs of animals infected with the highly pathogenic H5N1 avian influenza virus.^28^

**Table 3:** Evolution of swine influenza A virus.

1918-1919	H1N1 pandemic also affected swine
1930	The first isolation of H1N1 in pigs
1968	H3N2 infect swine in Asia after human pandemic
1976	Outbreak of new H1N1 swine strain of A/New Jersey/1976 occurred in military personnel at Fort Dix, New Jersey
1998	Triple reassortant viruses were isolated from pigs
1958-2005	37 human swine-origin influenza were reported
2005-2009	11 sporadic triple reassortant swine influenza viruses were reported in human
2009	New quadruple reassorted swine influenza H1N1 strain(A/California/07/2009)emerged in human populations and caused global influenza pandemic

### Epidemiology and impact

Epidemiological data now indicate that the 2009 H1N1 influenza virus pandemic started as an outbreak of influenza like illness in the Mexican town of La Gloria, Veracruz, in mid-February 2009. In mid-April the Center of Disease Control (CDC) identified swine origin H1N1 influenza virus in two specimens independently collected in southern California. By the end of April, international spread and human-to-human transmission prompted the WHO to increase the pandemic alert from Phase 3 to Phase 4 and shortly after to Phase 5. On June 11, 2009, the WHO raised its pandemic to the highest level Phase 6, indicating widespread community transmission on at least two continents (Table 4).^30^–^35^ As of December 27, 2009, more than 208 countries and overseas territories/communities have each reported at least one laboratory-confirmed case of pandemic H1N1 influenza, with a total or more than 622 000 laboratory confirmed cases and at least 12 220 deaths. However, the number of cases reported vastly underestimates the real number of cases: the WHO ceased regular reporting of case counts on July 16, 2009 because many countries were having difficulty tracking their numbers, and the WHO judged that their time would be better spent on investigating severe cases and other exceptional events. Most patients in the world with 2009 H1N1 have been teenagers and young adults, with rates of hospitalization highest in very young children. Between 1% and 10% of clinical illness require hospitalization. Overall from 7% to10% of all hospitalized patients are pregnant women in their second or third trimester. Of the hospitalized patients from 10% to 25% have required admission to intensive care, and 2% to 9% have died.^36^ Little is known about the level of pre-existing immunity to the 2009 H1N1 virus. Recent studies suggest that persons under the age of 30 years have little evidence of protective antibodies. However, a portion of older adults have pre-existing cross-reactive antibodies, presumably as a result of exposure to H1N1 strains circulating before 1957.^37^,^38^ Transmission of 2009 H1N1 virus from person to person is similar to that of other influenza viruses. The main route of transmission is respiratory through inhalation of large-particle respiratory droplets, and possibly via droplet nuclei. Transmission via large-particle droplets requires close contact because these droplets do not remain suspended in the air and generally travel only short distances (less than 2 meters). Contact with contaminated surfaces is another possible source of transmission. All respiratory secretions and bodily fluids (e.g. fomites, diarrheal stool) of infected persons should be considered potentially infectious.^39^–^41^ The secondary attacks rate in households was estimated to be 27.3% and in school settings an infected school child was estimated to infect 2.4 other children within the school.^39^ The estimated incubation period could range from 1 to 7 days, but is most likely 1 to 4 days. Infected persons can be assumed to be shedding virus from one day prior to illness onset until resolution of symptoms (up to 7 days following illness onset). Children and immunocompromised or immunosuppressed persons may be contagious for longer periods. The amount of virus shed is greatest during the first 2 to 3 days of infection and appears to correlate directly with the height of fever.^42^ The 2009 pandemic H1N1 virus is expected to come in waves, and we are now in the middle of the second wave. This wave may continue during winter, or there may be a third wave.^43^ As of today no increase of severity has been seen and genetic mutations have been minimal.

**Table 4:** World Health Organization pandemic levels.

Phase 1	No viruses circulating among animals have been reported to cause infections in humans.

Phase 2	An animal influenza virus circulating among domesticated or wild animals is known to have caused infection in humans, and is therefore considered a potential pandemic threat.

Phase 3	An animal or human-animal influenza reassortant virus has caused sporadic cases or small clusters of disease in people, but has not resulted in human-to-human transmission sufficient to sustain community-level outbreaks. Limited human-to-human transmission may occur when there is close contact between an infected person and an unprotected caregiver, but the virus is not widely transmitted among humans.

Phase 4	Verified human-to-human transmission of an animal or human-animal influenza reassortant virus able to cause “community-level outbreaks”. The risk of pandemic is significantly raised.

Phase 5	Human-to-human spread of the virus into at least two countries in one WHO region. The declaration of Phase 5 is a strong signal that a pandemic is imminent

Phase 6	The pandemic phase is characterized by community level outbreaks in at least one other country in a different WHO region in addition to the criteria defined in Phase 5. A global pandemic is under way.

### Clinical features

The clinical manifestations can vary from asymptomatic infection to serious fatal illness that may include exacerbation of other underlying conditions or severe viral pneumonia with multi-organ failure. The Centers for Disease Control and Prevention (CDC) defines cases as influenza-like illness (ILI) if there is a fever of >37.8°C (>100°F) plus cough and/or sore throat in the absence of a known cause other than influenza. In the outbreak of 2009 H1N1 influenza pandemic in New York City, 95% of virologically proven cases satisfied the ILI definition.^40^ Fever has been absent in some outpatients and in up to 1 in 6 surviving hospitalized patient.^44^ Vomiting and or diarrhea have occurred in up to 38% of outpatients in United States.^32^,^44^ Young children may have atypical influenza illness with absence of fever and cough. Among 89 children with confirmed H1N1 who required hospitalization in Birmingham, UK, the most common symptoms were fever (81%), cough (73%), and diarrhea (62%).^45^ Infants may present with fever and lethargy. The CDC case definitions for confirmed, probable and suspected cases are presented in Table 5.

**Table 5:** CDC: Case definition for 2009 H1N1 influenza virus.

Confirmed case:	An individual with an acute febrile respiratory illness with laboratory confirmed 2009 H1N1 infection by one or more of the following tests:
	- Real time reverse-transcription polymerase (rRT-PCR), or - Viral culture

Probable case	An individual with influenza like illness (i.e. an illness with a fever and cough or sore throat) who is positive for influenza A, but negative for H1 and H3 by rRT-PCR

Suspected case	An individual who does not meet the definitions of confirmed or probable pandemic H1N1 influenza A, but has ILI an epidemiologic link (e.g. likely exposure to a confirmed or probable case within the past 7 days.

Three categories of clinical presentations have been seen during the current pandemic:^40^,^46^

1. Mild illness characterized by fever (some patients had no fever), cough, sore throat, diarrhea, myalgias, headache. Other frequent findings have included chills and malaise. Vomiting and diarrhea have been reported in some patients, but no shortness of breath, dyspnea, or severe dehydration.
2. Progressive illness characterized by mild illness in addition to signs or symptoms suggesting a progressive illness, which include (Table 6):

**Table 6:** Clinical signs indicating rapid progression and need for urgent medical care.

In adults	In children
- Difficult breathing or shortness of breath	- Tachypnea or labored breathing
- Pain or pressure in the chest or abdomen	- Skin color change, gray or blue
- Episodes of sudden dizziness	- Inadequate intake of oral fluids
- Severe or continuous vomiting	- Severe or continuous vomiting
- Influenza-like illness that improves but then returns with fever and cough	- Influenza-like illness that improves but then returns with fever and cough
- Confusion	- Irritable, or not waking up
  - Chest pain, tachypnea, or labored breathing in children
  - Hypotension
  - Confusion or altered mental status
  - Severe dehydration or exacerbations of a chronic conditions (e.g. asthma, cardiovascular conditions)
3. Severe illness characterized by the following:
  - Profound hypoxemia, abnormal chest radio graph, and mechanical ventilation
  - Encephalitis or encephalopathy
  - Shock, multisystem organ failure
  - Myocarditis and rhabdomyolysis
  - Invasive secondary bacterial infection (e.g. pneumococcal disease)

### Complications

Most patients appear to have mild illness and recover spontaneously. Approximately 2% to 5% of laboratory-confirmed 2009 A (H1N1) influenza in Canada and in the United States as well as 8% in Mexico have required hospitalization.^44^ Nearly three-quarters of cases in the USA requiring hospitalization, as well as 21 (46%) of 45 fatal cases in Mexico, involved one or more underlying conditions including asthma, diabetes, heart or lung disease, neurologic disease, pregnancy, morbid obesity, autoimmune disorders and associated immunosuppressive therapies.^44^,^46^,^47^ Forty-five percent of patients admitted to intensive care units in the USA series were children under the age of 18 years, and 5% were 65 years of age or older. Surveillance of pediatric deaths reported by CDC indicated that, of 36 children who died, seven (19%) were aged <5 years, and 24 (67%) had one or more high-risk medical conditions. Twenty-two (92%) of the 24 children with high-risk medical conditions had neurodevelopmental disabilities which included cerebral palsy, developmental delay, autism, congenital neurological disorders and other central nervous system disorders.^49^ Pneumonia is the most common and serious complication of the 2009 H1N1 pandemic influenza. The clinical course of 45 fatal cases in Mexico was characterized by severe pneumonia, hypoxemia with multifocal infiltrates including nodular alveolar, or basilar opacities on chest x-ray, and rapid progression to acute respiratory distress syndrome (ARDS) and renal or multi-organ failure. A similar experience was reported from Canada, Australia, and the New Zealand. Some patients who required intensive care required advanced mechanical ventilation with high-frequency oscillatory bilevel ventilation and mean airway pressures of 32 to 55 cm/H_2_O or veno-venous extracorporeal membrane oxygenation (ECMO) support.^50^–^54^ Bacterial co-infections likely played a role in almost one-third of fatal cases of 2009 pandemic influenza A (H1N1) in the USA. The CDC investigators found evidence of concurrent bacterial infection in lung specimens from 22 of 77 patients (29%) with fatal pandemic H1N1 infection. A total of 10 cases were co-infections with *Streptococcus pneumoniae*, 6 with *Streptococcus pyogenes*, 7 with *Staphylococcus aureus*, 2 with *Streptococcus mitis* and 1 with *Hemophilus influenza*. Four of the fatal cases involved multiple pathogens. The age of patients ranged from 2 months to 56 years, with a median of 31 years.^55^ Among other complications of pandemic H1N1 are acute neurologic syndromes reported in four patients aged 7 to 17 years who were admitted with signs of ILI and findings that included seizures or altered mental status in 2 children, encephalitis in 2, and ataxia in 1. Three of the four patients had abnormal electroencephalogram (EEG). In all patients pandemic H1N1 viral RNA was detected in nasopharyngeal specimens but not in cerebrospinal fluids (CSF). All recovered without sequelae.^56^ The overall case-fatality rate was 0.4% (compared with 2.4% for the 1918-1918 influenza pandemic) based on surveillance data from Mexico and mathematical modeling.^57^,^58^ There was a documented underlying medical condition in at least 49% of global documented fatal cases.^58^

### Diagnosis

When influenza viruses are known to be circulating in the community, patients presenting with mild influenza can be diagnosed on clinical and epidemiological grounds alone. All patients should be instructed to return for follow-up should they develop any signs or symptoms of progressive disease (Table 6) or fail to improve within 72 hours of the onset of symptoms. Under no circumstances should influenza diagnostic tests delay initiation of infection control practices or antiviral treatment if 2009 H1N1 pandemic disease is suspected. Laboratory testing should be prioritized to include hospitalized patients; patients where a diagnosis of influenza will inform decisions regarding clinical care, infection control, or management of close contacts; and patients who have died of an acute illness in which influenza was suspected.

The gold standard for laboratory diagnosis of the 2009 H1N1 influenza is the real-time reverse transcriptase polymerase chain reaction (rRT-PCR) test, using primer and detector sequences tailored to the specific detection of this virus. A number of other diagnostic tests are available to detect the presence of 2009 H1N1 influenza in clinical specimens, but they differ in their sensitivity and specificity. Rapid influenza diagnostic tests are based on various forms of antigen detection and have high specificity (>95%) but variable sensitivity (10-70%).^59^–^60^ Preferred respiratory specimens include a nasopharyngeal swab with synthetic tip (e.g. polyester or dacron), nasal wash, bronchoalveolar lavage (BAL) or endotracheal aspirate. Lower respiratory tract specimens have a higher yield in patients with pneumonia due to viral replication in the lower respiratory tract. Many experts advise the use of a combination of nasopharyngeal swab with oropharyngeal swab. Isolation of H1N1 virus in cell culture or embryonated eggs is diagnostic for infection, but it may not a yield timely results for clinical management; in addition a negative viral culture does not exclude infection.^59^,^60^ All diagnostic laboratory work on clinical sample from patients who are suspected cases of influenza H1N1 virus infection should be done in a biosafety level 2 (BSL-2) laboratory. Growth of H1N1 virus in cell culture or embryonated eggs should be performed in a BSL-2 laboratory using BSL-3 practices.^60^

## Management of 2009 H1N1 influenza

The majority of individuals infected with the pandemic H1N1 influenza A virus can be treated with simple supportive care at home using antipyretics (e.g. acetaminophen or ibuprofen). Aspirin (acetylsalicylic acid) or aspirin-containing products (e.g. bismuth subsalicylate, Pepto-Bismol) should not be used in children <18 years due to the risk of Reye's syndrome.

Empiric antiviral therapy should be started as soon as possible for persons with suspected probable or confirmed influenza and:^45^

1. Illness requiring hospitalization
2. Progressive, severe or complicated illness regardless of previous health status and/or
3. High risk for severe disease (Table 7)

**Table 7:** High risk groups for severe illness.

Children younger than 2 years old
Pregnant woman up to 2 weeks post partum (regardless how the pregnancy ended)
Adult, 65 years of age or older
Persons younger than 19 years who are receiving long-term aspirin therapy.
Persons with medical condition including asthma, neurological and neurodevelopmental conditions (including disorder of the brain, spinal cord, peripheral nerve, and muscle such as cerebral palsy) chronic obstructive lung disease, cardiac disease, diabetes mellitus, immunosuppressive conditions (including HIV/AIDS, and cancer)

Recent reports have shown that 21% to 25% of hospitalized patients with confirmed 2009 H1N1 infections have not received antivirals or have delay in receiving antivirals.^47^,^48^ Among 27 fatal cases in Mexico, the median time from the appearance of symptoms to treatment with antivirals was 8 days (range, 1-26 days).^44^

## Antiviral drugs for treatment of 2009 H1N1 influenza (Table 8)

**Table 8:** Antiviral treatment and chemoprophylaxis of 2009 H1N1 influenza.

Medication/Age groups		Treatment (5 days)	Chemoprophylaxis (10 days)
**Oseltamivir**			

Adults		75 mg twice daily	75 mg once per day

Children (age≥12 months), weight	≤15 kg	30 mg twice daily	30 mg once per day
	15-23 kg	45 mg twice daily	45 mg once per day
	24-40 kg	60 mg twice daily	60 mg once per day
	>40 kg	75 mg twice daily	75 mg once per day

Children	Age 3 months to <12 months	3 mg/kg/dose twice daily	3 mg/kg/dose once per day
Children	0-<3 months	3 mg/kg/dose twice daily	Not recommended, unless situation judged critical (limited data)

**Zanamivir**			

Adults		Two 5-mg inhalations (10 mg total) twice daily	Two 5-mg inhalations (10 mg total) once daily

Children	≥7 years or older for treatment; ≥5 years for chemoprophylaxis	Two 5-mg inhalations (10 mg total) twice daily	Two 5-mg inhalations (10 mg total) once daily

The neuraminidase inhibitors, oseltamivir (Tamiflu) and zanamivir (Relenza) are the drugs of choice for treatment and while the vast majority of pandemic H1N1 circulating strains are sensitive to these medications, all strains tested are resistant to amantadine and rimantadine.^61^,^62^

Oseltamivir and zanamivir are generally well-tolerated. Nausea and vomiting were reported with moderate frequency among adults receiving oseltamivir for treatment (nausea without vomiting, 10%; vomiting 9%). In children treated with oseltamivir, 14% reported vomiting. Oseltamivir suspension is formulated with sorbitol, which may be associated with diarrhea and abdominal pain in patients who are fructose-intolerant. Zanamivir is formulated for oral inhalation and is contraindicated in patients with asthma or chronic obstructive disease. As of December 18, 2009, 136 isolates (among more than ten thousand tested) of pandemic H1N1 were resistant to oseltamivir.^62^ Among the 32 cases for whom detailed information was available, 16 were associated with antiviral prophylaxis, and three had no history of exposure to oseltamivir. Resistance was associated with the common H275Y mutation with retention of zanamivir susceptibility.^61^ Antiviral therapy is most effective when started within 48 hours after the onset of symptoms; however, evidence suggests that treatment^62^ may benefit patients with prolonged or severe illness even when started more than 48 hours after the onset of illness. The recommended duration of treatment is 5 days. Hospitalized patients with severe infection might require longer antiviral courses. Some experts have advocated use of doubled doses of oseltamivir in critically ill patient despite lack of published date about efficacy. Zanavimir inhaled formulation is not designed to be used in any nebulizer or mechanical ventilator^62^,^63^ as there is a risk that the lactose drug carrier can obstruct ventilator equipment. For patients who are unable to take oral medication or in whom oral medication appears to be ineffective, peramavir, an investigational neuraminidase inhibitor formulated for intravenous administration, can be requested from the CDC under Food and Drug Administration (FDA) and emergency use authorization, although studies on efficacy and safety are limited.^62^,^63^

Symptomatic patients who have highly suspected or documented oseltamivir resistance should not be treated with peramivir because strains with the H275Y mutation have demonstrated reduced in vitro susceptibility to peramivir. These patients should be treated with intravenous zanamivir, which is an investigational drug that can be requested from FDA for compassionate use.^62^,^63^ The CDC suggests limiting the use of antiviral chemoprophylaxis to specific groups. Antiviral doses recommended for treatment and prophylaxis of 2009 H1N1 influenza in adult and children are listed in Table 8. Clinicians should consider empiric treatment with antibacterial drugs if bacterial co-infections is suspected during or after influenza. Antibiotic selection should take into consideration local data regarding frequency of pathogens causing secondary infection and the pattern of drug resistance. When pneumonia is present, treatment with antibiotics should follow evidence-based guidelines for community-acquired pneumonia.

The use of corticosteroids for H1N1 influenza is controversial. High-dose systemic corticosteroids are not recommended for use in viral penumonitis outside clinical trials. However, low-dose steroids may be considered in patient with septic shock who require vasopressors.^44^,^64^,^65^

## Isolation of the hospitalized patient with 2009 H1N1 infection

CDC recommends standard, droplet, and contact precautions for care of patients with suspected or confirmed 2009 H1N1 influenza infection. Health care workers should use surgical masks for routine non-aerosolizing patient care and N95-respirators for aerosol-generating procedures. Isolation precautions should continue for 7 days after illness onset or until 24 hours after the resolution of fever and respiratory symptoms. A longer period of isolation may be considered in the case of young children and severely immunocompromised patients.^60^,^66^

## 2009 H1N1 vaccine

An effective vaccine is the best tool to prevent the unpredictable spread of the current influenza pandemic. The 2009 H1N1 virus has the potential to cause severe disease, death, and potential socioeconomic dysfunction, and mathematical modeling suggests that the effect of the virus can be reduced by immunization.^67^,^68^ Two types of H1N1 vaccines which have been prepared and have received approval from the FDA or the European Medicine Agency (EMEA) for use in the prevention of influenza caused by the 2009 pandemic influenza A (H1N1) virus. Both adjuvanted and unadjuvanted vaccine formulations are available. An adjuvant is a substance that boosts the immune response. It is made up of naturally occurring oil, water and vitamin E. The unadjuvanted vaccine does not include this material. Vaccination campaigns are currently underway to protect populations from pandemic H1N1. Preliminary data indicate that both vaccines are safe and immunogenic.^69^,^71^–^73^ The Advisory Committee on Immunization Practice (ACIP) recommends that vaccination efforts should focus initially on persons in five target groups at high risk for influenza-related complications (Table 9).^70^

**Table 9:** ACIP priority target groups for H1N1 influenza vaccine

Pregnant woman
Household contact and caregivers for infant younger than 6 months of age
Health-care and emergency medicine personnel
All people from 6 months through 24 years of age
Persons aged 25 through 64 years who have health conditions associated with high risk of medical complications from influenza (Table 7)

People who have had egg allergies should not receive the H1N1 vaccine without first consultation a physician because the viruses for the vaccine are grown in chicken-egg-based cultures. On November 19, 2009, the WHO estimated that around 80 million doses of pandemic vaccine had been distributed globally and around 65 million people had been vaccinated. The side-effect profile of the H1N1 vaccine (adjuvanted and unadjuvanted) particularly the frequency and severity of solicited adverse events is consistent with previous experience with seasonal influenza vaccine. To date, less than ten suspected cases of Guillain-Barré syndrome have been reported in people who have received vaccines. These numbers are in line with normal background rates of this illness as recently reported. All such cases are been investigated to determine whether these are randomly occurring events or whether they might be associated with vaccination.^69^ WHO has received no reports of fatal outcome or confirmed cases of Guillain-Barré syndrome since the H1N1 vaccination campaigns began. All cases have recovered. Intense active monitoring for rare adverse reactions of H1N1 vaccine is ongoing, but all data compiled to date indicate that pandemic H1N1 vaccines match the excellent safety profile of the seasonal influenza vaccines that has been used for more than 60 years.^69^,^70^
